# D2‐resected stage IIIc gastric cancer patients benefit from adjuvant chemoradiotherapy

**DOI:** 10.1002/cam4.873

**Published:** 2016-09-26

**Authors:** Jin Peng, Yuehua Wei, Fuxiang Zhou, Jing Dai, Yahua Zhong, Conghua Xie, Yue'e Qin, Jun Gong, Bin Xiong, Yunfeng Zhou

**Affiliations:** ^1^ Department of Medical Oncology & Radiation OncologyZhongnan Hospital of Wuhan UniversityWuhanHubei430071China; ^2^ Hubei Cancer Clinical Study CenterWuhanHubei430071China; ^3^ Department of Surgical OncologyZhongnan Hospital of Wuhan UniversityWuhanHubei430071China

**Keywords:** Adjuvant, chemoradiotherapy, lymph node excision, overall survival, stomach neoplasms

## Abstract

Although adjuvant chemoradiotherapy has been an important part in the treatment of gastric cancer, whether or not adjuvant radiation can benefit patients undergoing resection with D2 lymph node dissection remains controversial. This retrospective study aimed to evaluate the role of adjuvant chemoradiotherapy on patients with D2‐resected gastric cancer. A total of 337 patients with resected gastric cancer treated at Zhongnan Hospital of Wuhan University from 2004 to 2012 were retrospectively analyzed. Eligible patients were divided into the adjuvant chemoradiotherapy group (CRT;* n* = 124) and the adjuvant chemotherapy group (CT;* n* = 213). The primary endpoints were disease‐free survival (DFS) and overall survival (OS), with toxicity as the secondary endpoint. A subgroup analysis was performed based on clinical staging. The two groups were comparable in baseline characteristic, except for the number of lymph nodes dissected. The median OSs in the CRT and CT groups were 51.0 months and 48.6 months, respectively (*P *=* *0.251), and the median DFSs were 40.7 months and 31.2 months, respectively (*P *=* *0.112). Subgroup analysis revealed that the median OSs in patients at stage IIIc in the CRT group and CT group were 29.0 and 23.0 months, respectively (*P *=* *0.049), and those of the median DFSs were 21.2 and 15.1 months, respectively (*P *=* *0.015). There was no significant difference in main adverse events between two groups. Collectively, adjuvant chemoradiotherapy in gastric cancer patients with D2 resection was well tolerated. For Stage IIIc patients, the addition of adjuvant chemoradiotherapy was associated with a significant benefit in both OS and DFS.

## Introduction

Gastric cancer is the fourth most common cancer and the second leading cause of cancer‐related deaths worldwide. Approximately one million people develop gastric cancer and more than 700,000 people die from it every year 1. Surgical resection is the primary treatment modality and the only chance for a cure. However, surgery alone often results in high rates of both local recurrence and distant metastasis. Even after a complete surgical resection, many patients, particularly those with advanced disease, will relapse and eventually die from disease progression, which makes adjuvant treatment a priority for these patients 2, 3.

Several clinical trials have established the role for adjuvant therapies in the treatment of gastric cancer. The Intergroup (INT) 0116 trial was the first phase III clinical trial to demonstrate the benefit of adjuvant chemoradiotherapy as compared with surgery alone 4. This trial led to the use of adjuvant chemoradiotherapy as a part of the standard therapy in the USA. However, most of the patients enrolled in INT 0116 received D0 or D1 lymph node dissections, which are associated with a higher local recurrence rate as compared with D2 resections. Therefore, whether or not adjuvant radiation can benefit patients undergoing resection with D2 lymph node dissection remains controversial after INT trial. The ARTIST trial was the largest randomized clinical trial addressing this issue 5. Even after a 7‐year follow‐up, this trial did not identify a benefit from adjuvant chemoradiotherapy over adjuvant chemotherapy in the group as a whole, but in two unplanned subgroup analyses of higher positive node ratio and intestinal‐type gastric cancer patients, there was a significant improvement in disease‐free survival (DFS) and overall survival (OS). However, the controversy continued after this trial because 60% of the patients enrolled had early‐stage disease (stages Ib/IIa) and would not typically be considered the candidates for adjuvant chemotherapy 6. Moreover, most gastric cancer patients worldwide are initially diagnosed at the advanced or locally advanced stage, whereas in South Korea, where the ARTIST trial was conducted, they are frequently diagnosed at an earlier disease stage. Thus, the result of ARTIST may not be fully applicable in the USA or China, where most patients are diagnosed at locally advanced stage.

In China,gastric cancer is ranked No. 2 in the incidence rate as well as the cancer‐related mortality in all types of cancer. Most of gastric cancer patients are usually diagnosed in the locally advanced or advanced stage. A total of 498,000 patients died from gastric cancer in 2015 7, and for stage N3 patients, the 5‐year survival rate was only 3.1–8.2% 8. Considering the serious threat posed by the locally advanced gastric cancer, research on the adjuvant treatment of gastric cancer is of a particular concern in China.

For these reasons, we conducted the retrospective study to preliminarily evaluate the effects of adjuvant chemoradiotherapy on D2‐resected gastric cancer patients and to identify the subpopulations of Chinese patients who may benefit from adjuvant chemoradiotherapy. The results of this research may provide us with a valuable insight into which specific subpopulations of gastric cancer patients in the Chinese population should receive adjuvant treatment.

## Patients and Methods

### Patients

The medical records of gastric cancer patients who underwent D2 resection at the Zhongnan Hospital of Wuhan University (Hubei, China) from January 2004 to December 2012 were reviewed. All the clinical and follow‐up information was collected retrospectively. The Ethics Committee of Zhongnan Hospital approved the study protocol in April 2014, and a waiver of informed consent was obtained.

The main inclusion criteria were as follows: (1) histologically confirmed gastric adenocarcinoma, curative gastrostomy with D2 lymph node dissection, and R0 resection from January 2004 to December 2012; (2) patients aged between 18 and 75 years at diagnosis; (3) Stages T3 to T4 and/or N1 to N3 (2010 AJCC staging system 7th edition)^9^; (4) ECOG performance status of 0–2; and (5) adequate bone marrow function (hemoglobin≥90 g/L, neutrophil count≥1.5 × 10^9^/L, and platelet count≥100 × 10^9^/L); adequate liver function (serum bilirubin≤1.5 × ULN, aspartate transaminase and alanine transaminase≤3 × ULN); and adequate renal function (serum creatinine ≤0.106 mmol/L, and calculated creatinine clearance ≤50 mL/min).

The main exclusion criteria included the following: (1) D0 or D1 lymph node dissection; (2) receiving neoadjuvant treatment; (3) hepatic, renal, pulmonary, or cardiac dysfunction; (4) severe comorbidities, such as uncontrollable diabetes mellitus, uncontrollable hypertension, myocardial infarction, or unstable angina pectoris within 6 months of operation; (5) severe postoperative complications, such as anastomotic fistula and pancreatic fistula; and (6) diagnosis with another carcinoma before operation.

A total of 337 patients were eligible and enrolled in our study. These patients were divided into two groups according to the adjuvant treatment they received: the chemoradiotherapy group (CRT group) and chemotherapy group (CT group). There were 124 patients in the CRT group and 213 in the CT group. The median age was 54 (ranged 23–70) in the CRT group and 56 (ranged 25–70) in the CT group (*P* = 0.247). The main baseline characteristics of the patients enrolled are listed in Table 1.

**Table 1:** Patients’ demographics and clinical characteristics

	CRT Group(*N* = 124)	CT Group(*N* = 213)	*P* value
Age			0.247
Median	54	56	
Sex			0.287
Men	75 (60.4%)	145 (68.1%)	
Women	49 (39.6%)	68 (31.9%)	
ECOG Score			0.757
0	42 (33.9%)	64 (30.0%)	
1	78 (62.9%)	141 (66.2%)	
2	4 (3.2%)	8 (3.8%)	
Tumor location			0.964
Proximal	22 (17.7%)	41 (19.2%)	
Body	33 (26.6%)	52 (24.4%)	
Antrum	67 (54.0%)	117 (54.9%)	
Multiple/diffuse	2 (1.7%)	3 (1.5%)	
Histologic Grade			0.128
G1	4 (3.2%)	2 (0.9%)	
G2	23 (18.5%)	50 (23.5%)	
G3	87 (70.2%)	133 (62.4%)	
G4	10 (8.1%)	28 (13.2%)	
Staging			0.507
IIA	11 (8.9%)	17 (8.0%)	
IIB	34 (27.4%)	48 (22.5%)	
IIIA	25 (20.2%)	49 (23.0%)	
IIIB	21 (16.9%)	42 (19.7%)	
IIIC	33 (26.6%)	57 (26.8%)	
No. of lymph nodes dissected			0.038
Median	17	18	
Range	5–66	3–68	

CRT, chemoradiotherapy; CT, chemotherapy.

### Surgery

All the patients had undergone curative resection with extensive (D2) lymph node dissection. This procedure entailed resection of the perigastric lymph nodes and celiac, splenic or splenic‐hilar hepatic arterial lymph nodes, depending on the location of the primary tumor. The operations were performed by proficiently trained oncologists with corresponding certification in Zhongnan Hospital of Wuhan University.

### Adjuvant chemotherapy

Adjuvant chemotherapy was given 4–6 weeks after surgery. The cumulative number of chemotherapy cycles was 4–6 for FOLFOX regimen and six for the other chemotherapy regimen. For the CRT group, the first two cycles of chemotherapy were delivered before radiotherapy, while the remaining 2–4 cycles were delivered after radiotherapy. The following chemotherapy regimens were accepted in our study:(1) FOLFOX, oxaliplatin 85 mg/m^2^ intravenous (iv) on day 1, leucovorin 400 mg/m^2^ iv on day 1, 5‐FU 400 mg/m^2^ iv on day 1, and continuous iv 2500 mg/m^2^ in 46 h, repeated every 2 weeks; two times of chemotherapy counted as one cycle; (2) CAPOX, capecitabine 2000 mg/m^2^_*_day oral administration (po) on days 1–14 and oxaliplatin 135 mg/m^2^ iv on day 1, repeated every 3 weeks; (3) capecitabine or S1 monotherapy, capecitabine 2500 mg/m^2^_*_day po on days 1–14, repeated every 3 weeks or S1 80 mg/m^2^_*_day po on days 1–14, repeated every 3 weeks; and (4) capecitabine or S1 combination with cisplatin, capecitabine 2000 mg/m^2^*day po on days 1–14 or S1 60 mg/m^2^_*_day po on days 1–14 in combination with cisplatin 60 mg/m^2^ iv on day 1, and repeated every 3 weeks. These chemotherapy regimens are widely used in adjuvant chemotherapy for gastric cancer.

### Adjuvant radiotherapy and concurrent chemotherapy

Radiation was started 3–4 weeks after the second cycle of chemotherapy. Patients were treated in the supine position with 15 MV photons. The 3D‐conformal radiation therapy technique was used, and the dose delivered was 45 Gy, with 1.8 Gy daily fractions administered over 5 weeks. The radiation field consisted of the anastomosis site, duodenal stump, regional lymph nodes, and more than 2 cm from the margins of resection. The remnant stomach was not routinely included within the radiation field. The concurrent chemotherapy regimen was the same as that used in INT 0116 (intravenous fluorouracil 425 mg/m^2^ + leucovorin, 20 mg/m^2^ on the first 4 and the last 3 days of radiotherapy).

### Endpoints

The primary endpoints were DFS and OS with toxicity as the secondary endpoint. DFS was defined as the time from surgery to recurrence, second primary cancer, or death, whichever occurred first. OS was calculated from the date of surgery to the date of death from any cause or the last follow‐up.

### Follow‐up

Follow‐up assessments were performed every 3 months for the first 2 years after surgery and then every 6 months until the patient's death. Acute treatment‐related toxicities were documented according to Common Terminology Criteria for Adverse Events (version 4.0) 10 and the late toxicities were scored according to the RTOG/EORTC Late Radiation Morbidity Scoring Schema 11. The survival status of patients was ascertained in June 2014.

### Statistical analysis

Survival rate was calculated with the Kaplan–Meier method, and differences were expressed at the 5% significance level, using a two‐tailed log‐rank test. Analyses were performed using the SPSS 19.0 software (International Business Machines Corporation, Armonk, NY, United States).

## Results

The clinical characteristics of the recruited patients are listed in Table 1. The basic characteristics between the two groups were comparable, except for the number of lymph nodes dissected, which was higher in the CT group than in CRT group.

Seventy‐seven percent (77.4%) of the patients in the CRT group received chemotherapy as planned as compared with 81.2% in the CT group (*P *=* *0.402). FOLFOX was the primary chemotherapy regimen delivered in both groups. The profiles of the chemotherapy regimens delivered are listed in Table 2. The distributions of chemotherapy regimens delivered in the two groups were comparable. Ninety‐three percent (92.7%) of the patients in the CRT group had completed the 45 Gy of radiation as planned.

**Table 2:** Adjuvant chemotherapy regimen in the two groups

Regimen	CRT Group(*N* = 124)	CT Group(*N* = 213)	*P* value
FOLFOX	102 (82.2%)	167 (78.4%)	0.909
XELOX	7 (5.6%)	16 (7.5%)	
Capecitabine	8 (6.4%)	15 (7.0%)	
S1	5 (4.0%)	12 (5.6%)	
S1 + DDP	2 (1.8%)	3 (1.5%)	

CRT, chemoradiotherapy; CT, chemotherapy.

Grades 3–4 adverse events are documented in Table 3. The most frequent adverse events in both groups were nausea, vomiting, and leukocyte/neutropenia. The incidence rates were similar between the two groups. A total of 36.3% of the patients in the CRT group and 31.0% in the CT group experienced Grades 3–4 adverse events (*P *=* *0.338). A total of 2 (1.6%) and 3 (1.4%) patients in the CRT and CT groups, respectively, experienced febrile neutropenia. No treatment‐related death or grades 3–4 late adverse event was observed in either group.

**Table 3:** Grades 3–4 adverse events in two groups

	CRT Group(*N* = 124)	CT Group(*N* = 213)	*P* value
At least one adverse event	45/36.3%	66/31.0%	0.338
Nausea	13/10.4%	18/8.4%	0.533
Vomiting	12/9.6%	16/7.5%	0.487
Diarrhea	3/2.4%	6/2.8%	0.879
Anorexia	8/6.5%	10/4.7%	0.489
Hand‐foot syndrome	3/2.4%	5/2.3%	1.000
Fever	5/4.0%	8/3.8%	0.899
Diarrhea	3/2.4%	6/2.8%	0.879
Peripheral neurotoxicity	10/8.1%	18/8.4%	0.901
Leukocyte/neutropenia	27/21.7%	31/14.6%	0.090
Thrombocytopenia	5/4.0%	12/5.6%	0.517
Anemia	2/1.6%	4/1.8%	0.577
ALT/AST increase	2/1.6%	3/1.4%	1.000
Electrolyte imbalance	6/4.8%	7/3.3%	0.614

CRT, chemoradiotherapy; CT, chemotherapy.

**Figure 1:**
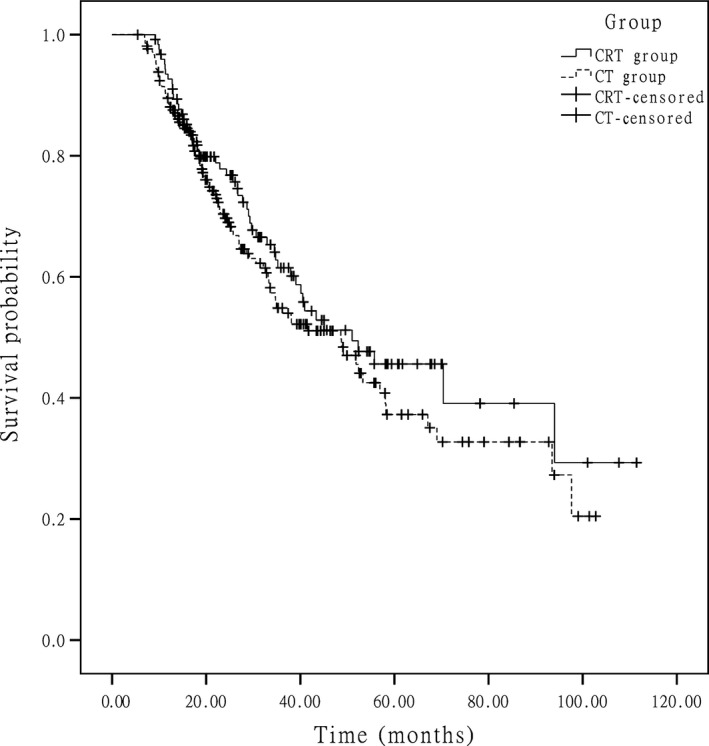
Kaplan–Meier estimated overall survival in the two groups.

**Figure 2:**
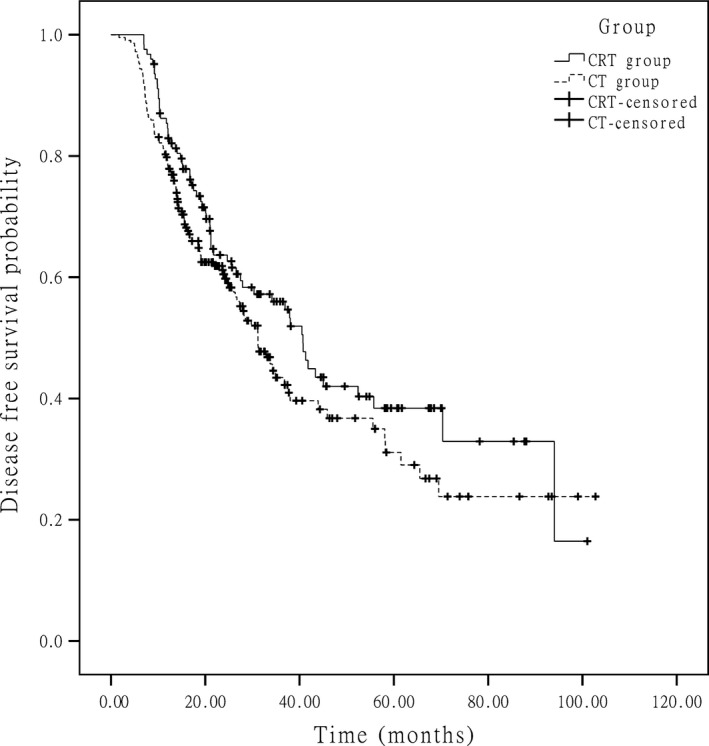
Kaplan–Meier estimated disease‐free survival in the two groups.

**Figure 3:**
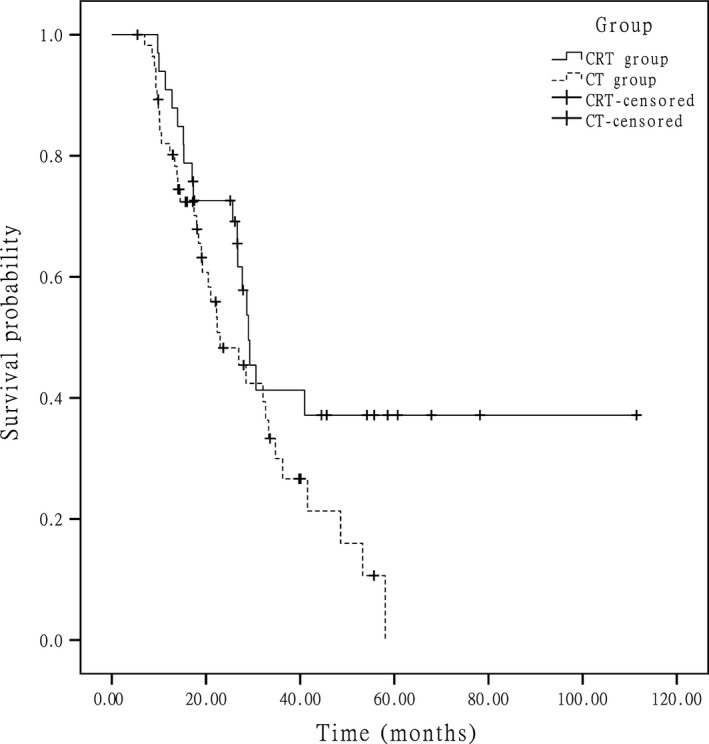
In stage IIIc patients, Kaplan–Meier estimated overall survival in the two groups.

**Figure 4:**
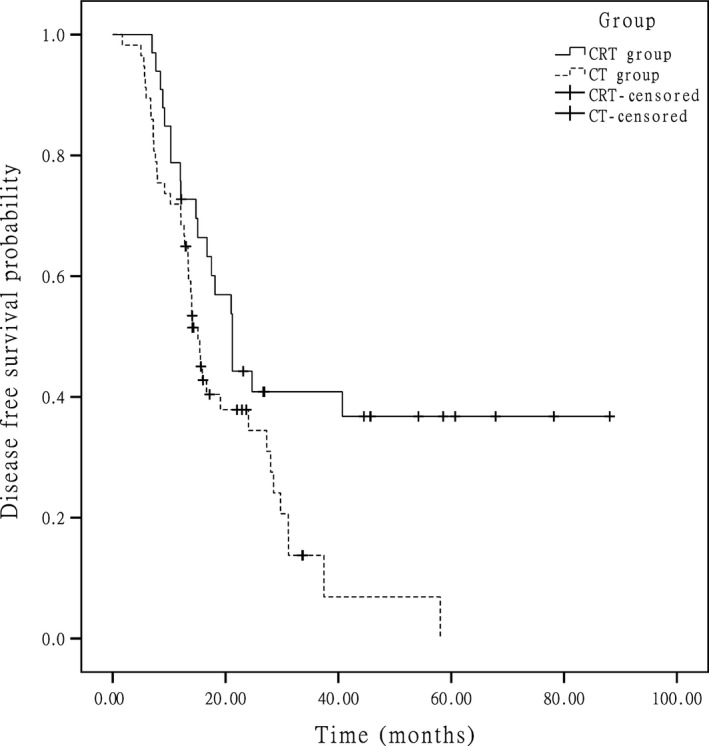
In stage IIIc patients, Kaplan–Meier estimated disease‐free survival in the two groups.

At the end of data accumulation in June 2014, the median follow‐up time was 41.1 months (range of 14–111.1 months) for the entire group. In the whole group, approximately 51.0% of the patients experienced recurrence, and 43.0% of the patients died. The median OSs were 51.0 months in the CRT group and 48.6 months in the CT group (*P *=* *0.251) (Fig. 1), respectively. The 5‐year survival rate was 45.6% in the CRT group and 37.3% in CT group (*P *=* *0.132). The median DFSs were 40.7 months in the CRT group and 31.2 months in the CT group (*P *=* *0.112) (Fig. 2), respectively. In the subgroup analysis for the patients at stage IIIc, the median OSs were 29.0 months in the CRT group and 23.0 months in the CT group and the difference between two groups was statistically significant (*P *=* *0.049) (Fig. 3), and the median DFSs were 21.2 months in the CRT group and 15.1 months in the CT group (*P *=* *0.015) (Fig. 4), respectively.

## Discussion

Radical gastrectomy with D2 lymph node dissection has been recommended as the standard surgical procedure for patients with gastric cancer in East Asia for several decades, and this procedure is increasingly accepted and recommended in the West 12, 13. However, whether or not the patients receiving D2 resection can benefit from adjuvant chemoradiotherapy remains controversial. Considering the increased control of local disease associated with D2 resection, the additional benefit of adjuvant chemoradiotherapy as compared with chemotherapy alone can be questionable. While our research did not detect any advantage of adjuvant treatment with chemoradiotherapy over chemotherapy alone, we did find that patients with gastric cancer at stage IIIc, in particular, could benefit from chemoradiotherapy with a longer OS and DFS. These results suggest that a subpopulation of the selected patients may benefit from adjuvant chemoradiotherapy.

While our study did not demonstrate a statistically significant benefit in the adjuvant chemoradiotherapy group as a whole, survival benefit was detected in the subgroup analysis of the stage IIIc patients. It has been debated for a long time whether gastric cancer patients can benefit from adjuvant chemoradiation after D2 resection. There have been mainly three prospective clinical trials respecting on this issue 5, 6, 14, 15. Though none of them had detected the survival benefit in the whole group analysis, ARTIST trial, which is the largest trial addressing this issue, reported DFS and OS profit in the subgroup with higher positive node ratio and the intestinal‐type subgroup. Our result is largely consistent with that of ARTIST trial 5. The inconsistency in results may be explained by several reasons as follows. Firstly, the selection of the participated patients may greatly affect the results. Not all the patients would benefit from adjuvant chemoradiotherapy after D2 resection but only those with a high risk of recurrence may receive an advantage from additional radiation aside from adjuvant chemotherapy; Secondly, radiation technique may also affect the final result. Both ARTIST trial and our research chose three‐dimensional conformal radiotherapy (3D‐CRT); and thirdly, definition of target volume may also affect the result. Clinical target volume (CTV) in our study did not include the remnant stomach, which will be discussed below. Thus, the toxicity profile was favorable and compliance was improved, both of which can also result in the improved outcome.

In our study, the median OSs were 51.0 months in the CRT group and 48.6 months in the CT group (*P *=* *0.251). Compared with the INT 0116 trial, the OSs of both groups in our study were more favorable, although the difference in OS between the two groups was not statistically significant. The INT 0116 Trial was a milestone for the adjuvant chemoradiotherapy for gastric cancer. It reported a survival improvement in patients who received adjuvant chemoradiotherapy as compared with surgery alone 4, 16. The considerable difference in OS between the INT Trial and our study can likely be explained by the selection of different surgical approaches. All the patients in our study received standard D2 lymph node dissection as compared with only 10% in the INT study. D2 lymphadenectomy is associated with lower rates of locoregional recurrence and cancer‐related death as compared with D1 resection 12, 13. Approximately 85% of the patients enrolled in INT 0116 had positive lymph nodes; therefore, insufficient resection leading to the higher local recurrence likely contributed to the lower OS in both groups of this trial.

In our study, the median DFSs were 40.7 months in the CRT group and 31.2 months in the CT group, which were much lower than those in the ARTIST trial. Though the inclusion criteria and research design in our study were quite similar to those in the ARTIST trial, our research results were markedly different. The most significant difference was the patient's distribution of staging; there were much more locally advanced (stage III) gastric cancer patients in our study, which is more consistent with the actual conditions in China and some other countries as well. Because most gastric cancer patients in Korea are generally diagnosed in earlier stages of the disease; consequently, nearly 60% of patients enrolled in the ARTIST trial were at the earlier stages (e.g., stages Ib and IIa) 6. This may be the main reason why the DFS was more favorable in the ARTIST trial than that in our study.

Another noteworthy difference between the ARTIST trial and our study was the number of lymph nodes dissected, which was larger in the ARTIST Trial. Although D2 lymph node dissection was utilized in both studies, the median number of lymph nodes dissected was less than 20 in our study as compared with more than 40 in ARTIST study. According to surgical principle, at least 15 lymph nodes should be dissected for accurate staging 13, 17. Although the number of lymph nodes dissected qualified most of the patients in our study, the surgical quality of our study was inferior to that in the ARTIST Trial. Several studies reported the survival benefit with more lymph node dissected 18, 19. However, another research revealed that the number of 16 might be a threshold in predicting survival benefit from lymph node dissection 20. In the study, hazard ratio (HR) was improved as the number of lymph nodes was increased up to 16. However, this improvement could no longer be observed when the number was above 16. Thus, the difference in survival may not necessarily be as a result of less number of lymph node dissections in our study.

Our study indicated that patients had good tolerance and compliance with adjuvant chemoradiotherapy. However, significant toxicities associated with adjuvant chemoradiotherapy have been typically a serious concern. In the INT 0116 trial, more than half of the patients experienced adverse events of Grades III‐IV. Approximately 36% of the patients interrupted treatment because of the intolerable side effects and other reasons 4. In ARTIST trial, 48.4% of patients experienced Grade 3–4 neutropenia and 81.7% of patients completed the planned treatment in the chemoradiation group 6. Generally speaking, the toxic effect and patient compliance are more favor in ARTIST trial and our research than in INT 0116 trial. The reason may lie in the progress of radiation technology. Both ARTIST trial and our research used three‐dimensional conformal radiotherapy (3D‐CRT) instead of two‐field anteroposterior opposing radiation technique, which is associated with a larger irradiation volume and more serious toxicity to normal tissue as compared with multiple‐field conformal radiation. Furthermore,considering the lower recurrence rate of the remnant stomach, the clinical target volume (CTV) in our study did not include the remnant stomach, which was evaluated in a Korean trial 21. Therefore, the toxicity profile in our study was more favorable as compared with that in the INT 0116 trial, for both the early and the late adverse events.

The hematologic toxicity was much lower in our study than in ARTSIT trial, though the similar radiation technology was applied in both studies. Why the hematologic toxicity showed such a big difference between two studies? This difference may be explained by two reasons as follows. Firstly, the precise target volume delineation guideline that can be accepted by most oncologists is still lacking, which will result in the difficulty in comparing the treatment responses and toxicities in different institutions. Secondly, capecitabine regimen was applied in concurrent chemotherapy in ARTIST as compared with 5‐FU/CF regimen in our study. Capecitabine has been believed to have a lower hematologic toxicity and better patient compliance in colon cancer 22, 23. It does not necessarily mean that the toxicity of capecitabine is superior to 5‐FU/CF regimen in concurrent chemotherapy. It was reported that in another adjuvant chemoradiation of gastric cancer, chemotherapy with capecitabine caused a 50.2% Grade 3–4 hematotoxicity 24, while in some trials with 5‐FU/CF as concurrent chemotherapy, the Grade 3–4 hematotoxicity rates were in the range of 5.9–14.8% 15, 25, 26. In terms of hematotoxicity, concurrent capecitabine regimen may not be superior to 5‐FU/CF. Beside hand‐and‐foot syndromes; capecitabine‐based concurrent chemotherapy has been proved more diarrhea as compared with 5‐FU/CF 27. So, before the head‐to‐head comparison of clinical trial is conducted, it is difficult to determine which concurrent chemotherapy regimen is less toxic based on the current data. On the basis of our study, we considered that 5‐FU/CF‐based chemoradiation could be well tolerable in gastric cancer patients with D2 resection.

The major limitation of this study is the imbalance in selection of patients due to the retrospective analysis itself. It is difficult to avoid imbalance in selection of patients in a retrospective study, although we believed that the variations in the number of lymph nodes dissected had little impact on the final conclusions. Constrained by a limited number of eligible patients, we had difficulty in selecting a uniform adjuvant chemotherapy regimen as an inclusion criterion in our study, although the final distribution of chemotherapy treatments was balanced between two groups. We initially planned to analyze the recurrence patterns in the two groups because the difference in the failure mode would have given us much more information about the efficacy of adjuvant chemoradiotherapy. However, this analysis was abandoned because the recurrence data were insufficient. A further study will address this issue once a sufficient amount of data are accumulated.

Our study preliminarily evaluated the role of adjuvant chemoradiotherapy in a Chinese population. Adjuvant chemoradiotherapy in gastric carcinoma patients with D2 resection was well tolerated. For Stage IIIc patients, the addition of adjuvant chemoradiotherapy was associated with a clinical benefit in both OS and DFS as compared with adjuvant chemotherapy alone. Our study suggests a potential guideline for the adjuvant treatment of gastric cancer, that is, the high‐risk patients who can benefit the most from adjuvant treatment should receive adjuvant chemoradiotherapy while some low‐risk patients may consider of waiving radiation after adjuvant chemotherapy. Our results should be further tested in a prospective clinical trial.

## Conflict of Interest

None.
